# Clinical Cases

**Published:** 1883-06

**Authors:** J. A. Biegler

**Affiliations:** Rochester, N. Y.


### CLINICAL BUREAU.

### A CLINICAL CASE.

J. A. Biegler, M. D., Rochester, N. Y.

April 12th, 1882.—Mrs. T. L. T., a middle-aged lady, of general healthy appearance, physically well formed, apparently free from dyscrasia. She had suffered for several months from pain in right ovary, so acute at times that she was disabled from getting about. It always hurt her to move. No discharge of any kind. She looked so free from chronic ailments that I questioned her closely, and sat patiently hoping for some clew, but it looked for a time like a hopeless task, as she was *mentally too ugly* and *obstinate* to give the help I needed. Having a will of my own, and always determined to be the master, we had a long sitting, some of the time without either speaking. At last a question brought light on the case. She had a severe attack of rheumatism of the right wrist joint three months ago, and called on an old school physician, who, she said, went to a drug store and scientifically compounded a liniment which relieved her almost instantly, expressing herself as if it were the grandest thing ever accomplished. This led to the question: How soon after that relief did you experience this pain in the right side? The answer was, after a little hesitation, "Why, I believe right away; I think the same day." I gave her three doses of Bryonia$^{cm}$ night and morning, followed by Sacch. lact. for a week. I said to her at the time, "That scientific gentleman has succeeded in transposing the disease, and before you get well you will have it back in the wrist." At the end of the week she came in looking bright and *pleasant*, standing and walking erect, as she did not do before. "Well, how are you now?" Answer: "I have no pain in my side; it is all gone, but my wrist pains me awfully; still, I feel well for all that." Wishing to learn her a lesson, I did not give her anything, not even blanks, and requested her to call in a week, which she did, and then came perfectly well, and remains so. This lady's husband is an intelligent man, and appreciated pure Homœopathy. I therefore asked her why she did not come to me on the first time when her wrist was affected. Her answer was that she thought I would ask her too many questions.

*March* 19*th*, 1883.

